# A molecular switch in RCK2 triggers sodium-dependent activation of K_Na_1.1 (KCNT1) potassium channels

**DOI:** 10.1016/j.bpj.2024.04.007

**Published:** 2024-04-10

**Authors:** Bethan A. Cole, Antreas C. Kalli, Nadia Pilati, Stephen P. Muench, Jonathan D. Lippiat

**Affiliations:** 1 School of Biomedical Sciences, University of Leeds, Leeds, United Kingdom; 2 Leeds Institute of Cardiovascular and Metabolic Medicine, University of Leeds, Leeds, United Kingdom; 3 Astbury Centre for Structural and Molecular Biology, University of Leeds, Leeds, United Kingdom; 4 Autifony Srl, Padova, Italy

## Abstract

The Na^+^-activated K^+^ channel K_Na_1.1, encoded by the *KCNT1* gene, is an important regulator of neuronal excitability. How intracellular Na^+^ ions bind and increase channel activity is not well understood. Analysis of K_Na_1.1 channel structures indicate that there is a large twisting of the βN-αQ loop in the intracellular RCK2 domain between the inactive and Na^+^-activated conformations, with a lysine (K885, human subunit numbering) close enough to potentially form a salt bridge with an aspartate (D839) in βL in the Na^+^-activated state. Concurrently, an aspartate (D884) adjacent in the same loop adopts a position within a pocket formed by the βO strand. In carrying out mutagenesis and electrophysiology with human K_Na_1.1, we found that alanine substitution of selected residues in these regions resulted in almost negligible currents in the presence of up to 40 mM intracellular Na^+^. The exception was D884A, which resulted in constitutively active channels in both the presence and absence of intracellular Na^+^. Further mutagenesis of this site revealed an amino acid size-dependent effect. Substitutions at this site by an amino acid smaller than aspartate (D884V) also yielded constitutively active K_Na_1.1, and D884I had Na^+^ dependence similar to wild-type K_Na_1.1, while increasing the side-chain size larger than aspartate (D884E or D884F) yielded channels that could not be activated by up to 40 mM intracellular Na^+^. We conclude that Na^+^ binding results in a conformational change that accommodates D884 in the βO pocket, which triggers further conformational changes in the RCK domains and channel activation.

## Significance

Sodium-activated potassium channels regulate neuronal excitability, and their dysfunction causes severe childhood disorders. Here, we identify a structural determinant in the intracellular domains that is responsible for triggering channel activation in response to sodium ion binding. An increase in the size of a particular amino acid renders the channel sodium insensitive, while a decrease in size enables the channel to activate in the absence of sodium. This enhances our understanding of how this subclass of potassium channels respond to changes in the intracellular ionic environment. Furthermore, this may also further our understanding of the basis of human neurological disorders and their treatment.

## Introduction

Na^+^-activated K^+^ (K_Na_) channels open in response to elevation in the cytoplasmic Na^+^ concentration, contributing to hyperpolarization of the membrane potential of neurons and other cell types. K_Na_1.1 and K_Na_1.2 (or SLACK and SLICK) are members of the large-conductance, regulator of K^+^ conductance (RCK) domain-containing subfamily of K^+^ channels and are activated by intracellular Na^+^ (1). Intracellular Na^+^ is an essential determinant of wild-type (WT) K_Na_1.1 channel opening, and in normal physiology, K_Na_1.1 activity is coupled to persistent inward Na^+^ currents, for example, through re-opening voltage-gated Na^+^ channels or NALCN channels that increase local Na^+^ concentration above that of the bulk cytosol (2,3). Additionally, transient currents through AMPA receptors located in the vicinity of K_Na_1.1 have also been implicated as a Na^+^ source for the channel as part of a negative feedback loop (4). The importance of these channels is highlighted by the seizure disorders and intellectual disability caused by pathogenic variants in either of the K_Na_-encoding genes, *KCNT1* or *KCNT2* (5,6,7,8,9). In most cases, pathogenic variants in either gene result in a missense mutation that leads to enhanced K_Na_ channel activity, but loss of function is also found with some *KCNT2* variants (10). Quinidine, bepridil, and clofilium were the first drugs to be identified as K_Na_ channel inhibitors, with bithionol, riluzone, loxapine, and niclosamide as activators (11,12,13). Each of these drugs is nonselective and unlikely to be of clinical use in K_Na_ disorders. In response to *KCNT1* gain-of-function disorders, several groups have identified novel K_Na_1.1 channel inhibitors (14,15,16). Our approach exploited K_Na_ channel structures and targeted the pore domain in virtual high-throughput screening (14), but it is conceivable that future structure-based screening could instead target the Na^+^-activation mechanism, which could improve specificity. However, the structural basis of how Na^+^ ions interact with K_Na_ channels and how this results in channel activation remains unknown.

Previously, residues located in the rat K_Na_1.1 RCK2 domain were proposed to form a Na^+^-binding site based upon a Na^+^ coordination motif, DXRXXH, that is found in Na^+^-activated GIRK channels (17). Mutating D818 and, to a lesser degree, H823 diminished rat K_Na_1.1 Na^+^ activation when the channels were expressed and recorded from patches excised from *Xenopus* ooctyes. In general, Na^+^ sensitivity was shifted to higher concentrations for channels mutated at either site (17). In human K_Na_1.2, mutation of the equivalent aspartate residue, D757, to arginine abolished Na^+^ activation in whole *Xenopus* oocytes, with function rescued by application of the activator niflumic acid (18). The structures of the chicken K_Na_1.1 channel in the closed (zero Na^+^ conditions) and activated (high Na^+^ conditions) conformations have since been resolved by cryo-electron microscopy (cryo-EM) and single-particle averaging (19,20), in which the Na^+^-binding fold proposed by Zhang and others (17) was not evident. The region containing the proposed DXRXXH coordination motif is conserved between chicken and rat K_Na_1.1 but remains as a static loop in both the active and inactive conformations, as will be described below. Since Na^+^ ions, owing to their size, are usually unresolved in structures generated by cryo-EM, it is not known where in the channel they bind and how this results in increased channel activity.

In studying the cryo-EM structures of the inactive and active chicken K_Na_1.1 channel subunits, we identified a conformational difference between the two that we hypothesized could underlie Na^+^-dependent activation. Furthermore, it appeared to involve the aspartate residue identified by Zhang and others (17) in playing a conformation-stabilizing role. Using site-directed mutagenesis and electrophysiology, we identify additional residues in a nearby region that are critical for Na^+^-dependent activation and propose that a twisting of a loop between βN and αQ in RCK2 acts as a molecular switch that underlies K_Na_1.1 activation. While preparing this manuscript, complementary studies on K_Na_1.1 Na^+^ binding and activation were published, involving molecular dynamics simulations and mutagenesis (21) or cryo-EM (22), together with functional characterization. In presenting and discussing our findings, we highlight where our interpretation is consistent or conflicts with the conclusions of either of these studies.

## Materials and methods

### Analysis of protein structures

Structures of chicken K_Na_1.1 in the Na^+^-activated (PDB: 5U70) and Na^+^-free (PDB: 5U76) states (20) were analyzed and figures prepared using UCSF Chimera (23). Amino acid substitutions were introduced in silico using the Rotamer function and assessed using the Find Clashes/Contacts function. Initially, the rotamer with the highest probability, with respect to the chi parameters generated from the integrated Dunbrack 2010 library, was selected. If intramodel atomic clashes were obtained, then the next two rotamers in the probability ranking were evaluated. Molecular surfaces were calculated using the MSMS tool within Chimera (24).

### Molecular biology and transfection

The full-length pcDNA6-K_Na_1.1 mammalian expression plasmid that we described previously (14) was used in these studies. Mutations were designed and introduced by polymerase chain reaction using the New England Biolabs method and confirmed by sequencing (Genewiz, Takeley, UK). Due to the large size and high GC content of the insert, mutations were generated in a plasmid containing the SbfI/BsiWI restriction fragment and then subcloned into the corresponding sites in the pcDNA6-K_Na_1.1 construct. Chinese hamster ovary (CHO) cells were cultured in Dulbecco’s modified Eagle’s medium (Gibco, Paisley, UK) supplemented with 10% (v/v) fetal bovine serum, 50 U/mL penicillin, and 0.05 mg/mL streptomycin and incubated at 37°C in 5% CO_2_. Cells were cotransfected with WT or mutated pcDNA6-K_Na_1.1 together with pEYFP-N1 plasmid using Mirus TransIT-X2 reagent (Geneflow, Lichfield, UK). For electrophysiological experiments, cells were plated onto borosilicate glass cover slips and used 2–4 days later.

### Electrophysiology

All chemicals were obtained from Sigma-Aldrich (Gillingham, UK) unless stated otherwise. Micropipettes were pulled from thin-walled borosilicate glass (Harvard Apparatus, Kent, UK), polished, and gave resistances of 1.5–2.5 MΩ in the experimental solutions. The bath (extracellular) solution contained, in mM, 140 NaCl, 1 CaCl_2_, 5 KCl, 29 glucose, 10 HEPES, and 1 MgCl_2_ (pH 7.4) with NaOH. The 40 mM Na^+^ pipette (intracellular) solution contained, in mM, 130 K-gluconate, 30 NaCl, 29 glucose, 5 EGTA, and 10 HEPES (pH 7.3) with KOH. To obtain pipette solutions containing Na^+^ at 10 and 0 mM (nominally Na^+^ free), the NaCl was replaced by equimolar amounts of choline chloride. K_Na_1.1 activators loxapine succinate and niclosamide were prepared as 10 mM stock solutions in DMSO. The final drug concentrations were obtained by diluting the stock solution in the bath solution on the day of the experiment.

Currents were recorded from EYFP-fluorescing cells at room temperature (20°C–22°C) in the whole-cell patch-clamp configuration using an EPC10 amplifier (HEKA Electronics, Lambrecht, Germany), with >70% series resistance compensation (where appropriate), 2.9 kHz low-pass filtering, and 10 kHz digitization. Following the establishment of the whole-cell configuration, cells were held at −80 mV, and 400 ms voltage pulses from −100 to +80 mV in 10 mV increments were applied. With experiments that examine the effect of pharmacological activation, the 10 mM NaCl pipette solution was used, and the voltage protocol was applied both before and after bath perfusion of 30 *μ*M of either niclosamide or loxapine.

### Data analysis

Samples were not randomized, and the experiments were not blinded. Data are presented as mean ± SEM from *n* number of cells. Statistical analysis was performed using SPSS (IBM Analytics, Portsmouth, UK), with the chosen tests indicated in the figure legends; *p* < 0.05 was considered significant. Without a priori knowledge of effect sizes, power calculations were not conducted. Representative whole-cell current traces were plotted and residual capacitance spikes removed in OriginPro. Whole-cell current-voltage relationships were divided by whole-cell capacitance to give the current density (pA/pF). Reversal potentials were obtained by fitting the linear part of current-voltage relationships around the reversal potential using linear regression and determining the voltage at the zero-current level. Conductance values (*G*) at each voltage (*V*_*m*_) were obtained by dividing current amplitudes (*I*) by the driving force on K^+^ ions, calculated using the reversal potentials (*V*_*rev*_) obtained in individual recordings: *G* = *I*/(*V*_*m*_ − *V*_*rev*_). The conductance values were plotted against *V*_*m*_ and fitted with a Boltzmann function, *G* = (*G*_*max*_ − *G*_*min*_)/(1 + e^(*V*^_*m*_^−*V*^_*0.5*_^)/*k*^) + *G*_*min*_, which gave values for activation midpoint (*V*_*0.5*_), *G*_*max*_, *G*_*min*_, and the Slope factor (*k*). Data were normalized by dividing by *G*_*max*_ for each experiment. Reported *V*_*0.5*_ values were corrected for liquid junction potential error after the experiment. With *k* = *RT/zF*, the valence of the gating charge, *z*, was estimated.

## Results

### Identification of a putative Na^+^-binding site in K_Na_1.1 structural data

**Figure 1:**
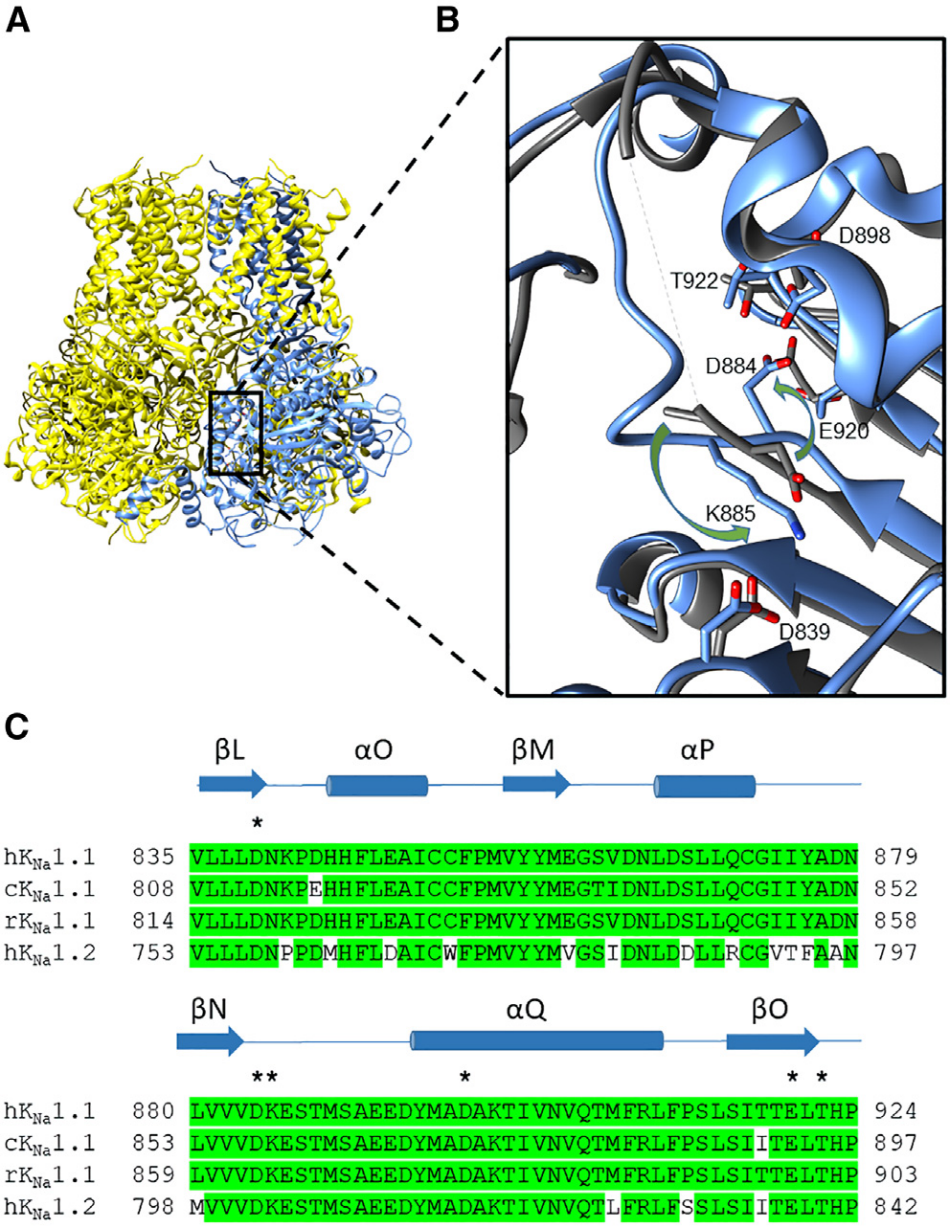
Structural analysis of the Na^+^-binding site proposed by Zhang and others (17) in chicken K_Na_1.1 channel protein resolved using cryo-EM. (*A*) Structure of chicken K_Na_1.1 in the active conformation (PDB: 5U70) with one subunit in the tetramer colored blue. Each subunit comprises six transmembrane helices (*top part of structure*) and a re-entrant P-loop between S5 and S6 that forms the K^+^-selective filter. The large intracellular domains contain a pair of RCK domains, RCK1 and RCK2 (*bottom part of structure*). The domain of interest in RCK2 is enclosed by the black box. (*B*) Enlarged representation of the domain of interest with the active (*blue*) and inactive (PDB: 5U76; *dark gray*) conformations overlaid. The aspartate identified by Zhang and others (17) is D839 (all human K_Na_1.1 numbering). The twisting of the loop between βN-αQ in RCK2, indicated by the green arrows, positions a lysine (K885) close enough to form a salt bridge with the purported Na^+^-binding aspartate (D839) in the Na^+^-activated state. Concurrently, an aspartate (D884) in the same loop adopts a position proximal to the βO strand and is flanked by E920 and T922. The complete βN-αQ loop is not resolved in the inactive state (*dashed line*), indicating disorder. (*C*) Sequence alignment of the domain of interest between K_Na_1.1 from human (h), chicken (c), and rat (r), plus the closely related human K_Na_1.2. Conserved residues (with respect to hK_Na_1.1) are shaded green. The domain structure (22) is shown by the blue shapes, and the amino acids labeled in (*B*) are indicated by asterisks.

The aspartate residue found by Zhang and others (17) to be critical for Na^+^ activation in rat K_Na_1.1, D818, is equivalent to D812 in chicken and D839 in human K_Na_1.1. To assist comparisons between studies of K_Na_1.1 clones from different species, the positions of the amino acids detailed in this study in human, rat, and chicken are provided in Table S1. We hereon refer only to the amino acid numbering in human K_Na_1.1. The proposed Na^+^-coordinating motif, based on that in GIRK channels, is conserved between species and forms the loop between βL and αO in the K_Na_1.1 RCK2 domain. Firstly, the Na^+^-binding fold proposed by the molecular modeling and simulations of RCK2 of the rat K_Na_1.1 (17) subunit was not observed in the cryo-EM structures of either the active (PDB: 5U70; Fig. 1 *A*) or inactive (PDB: 5U76) chicken K_Na_1.1 channels. Secondly, there was no discernible difference between the two structures in the positioning of the side chain of this aspartate or any of the neighboring residues previously proposed to contribute to Na^+^ binding (D839 in Fig. 1 *B*). Notably, in the structure of the Na^+^-activated state, a lysine reside in the loop between βN-αQ (K885 in Fig. 1 *B*) falls within 3.4 Å of the aspartate, sufficiently close to form a salt bridge. In the apo state, this loop is not fully resolved, indicating disorder, but comparison with the structure of the activated state suggests that upon activation by sodium, there is a rotation of this loop around the axis of the backbone by approximately 180°. This was observed by Zhang and others (22) to be the case in the structures of human K_Na_1.1. Consequentially, the adjacent aspartate in the same βN-αQ loop (D884 in Fig. 1 *B*) adopts a position within a pocket formed by the βO β-strand in RCK2 (Fig. 1 *B*). Specifically, the aspartate side chain occupies a crevice between the β-carbons of E920 and T922 side chains and the L921 backbone. The differences between the cryo-EM structures of chicken K_Na_1.1 in the inactive and Na^+^-activated states therefore indicate that the rotation and stabilization of the βN-αQ loop is a conformational change in RCK2 upon Na^+^ binding.

### Activation of WT K_Na_1.1 by intracellular Na^+^ and pharmacological activators

Guided by K_Na_1.1 channel structural data, a combination of mutagenesis and whole-cell electrophysiology was used to investigate the involvement of residues in Na^+^-dependent activation of the human K_Na_1.1 channel. In our hands, human K_Na_1.1 runs down within seconds in excised inside-out patches, leaving unitary or no currents. Therefore, to efficiently explore the effects on macroscopic K_Na_1.1 currents, we conducted whole-cell patch-clamp recordings from CHO cells expressing WT or mutant K_Na_1.1 using pipette solutions with different concentrations of Na^+^ and pharmacological activation to bypass Na^+^ activation and confirm the presence of relatively Na^+^-insensitive channels. The antipsychotic drug loxapine and the antihelminthic drug niclosamide are potent activators of WT K_Na_1.1 with half-maximal activation concentration (EC_50_) of 4.4 and 2.9 *μ*M, respectively (13). Both drugs also reduce the voltage dependence of K_Na_1.1 activation, resulting in near-linear current-voltage relationships and increased inward current at voltages negative to the reversal potential (Fig. S1). Initially, niclosamide was selected to confirm function expression of “inactive” mutant channels. CHO cells are believed to have little or no endogenous ion conductance (25). However, when 30 *μ*M niclosamide was perfused into the bath, a current with density 58.96 ± 9.60 pA/pF at +10 mV was recorded from nontransfected CHO cells, compared to 1.49 ± 0.67 pA/pF at +10 mV prior to its application (*n* = 4 and 5 cells, respectively). Though this current is relatively small in comparison to the exogenous K_Na_1.1 current (Fig. S1, *A* and *B*), this could confound the functional rescue of seemingly inactive K_Na_1.1 channels. The identity of the conductance and charge carrier evoked by niclosamide is unknown, but experiments ruled out K_Na_1.1, since the current was not inhibited by 10 *μ*M bepridil (Fig. S1 *C*). The reversal potential (−73.30 ± 0.85 mV, *n* = 4) would be consistent with a K^+^-selective conductance, but no further experiments were conducted to characterize the current and its ion selectivity. Loxapine had no effect on the membrane conductance of nontransfected CHO cells (Fig. S1, *A* and *B*) and was therefore more suitable for these experiments.

**Figure 2:**
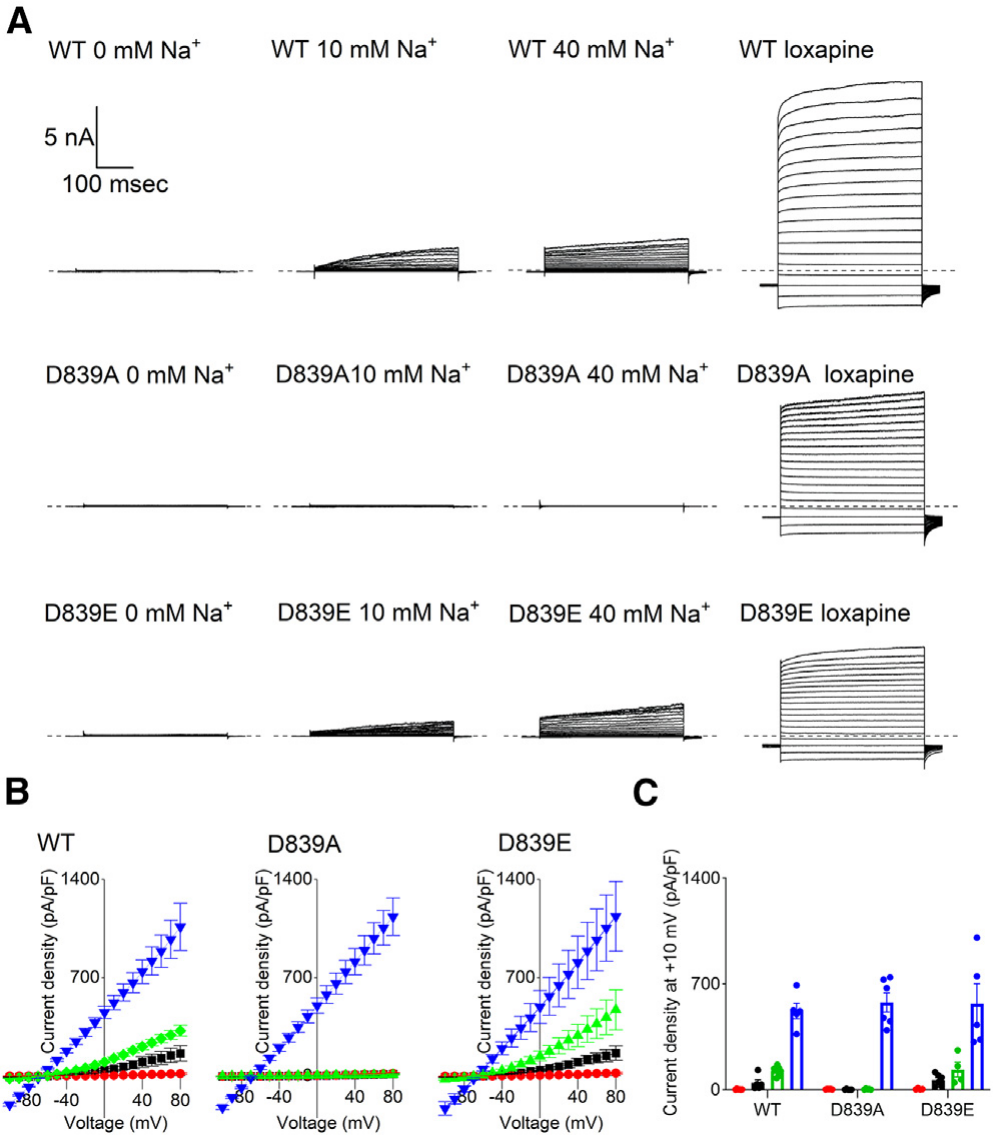
Mutational analysis of the previously proposed Na^+^ sensor. (*A*) Representative whole-cell currents from CHO cells transfected with WT or mutant human K_Na_1.1, with intracellular Na^+^ and drug exposure as indicated, in response to 400 ms steps from −100 to +80 mV in 10 mV increments from a holding potential of −80 mV. The dashed lines indicate the zero-current level. (*B*) Mean (±SEM, *n* = 5–8 cells) current-voltage relationships for WT and mutant K_Na_1.1 channels in the presence of 0 (*red circle*), 10 (*black square*), and 40 (*green diamond or green triangle*) mM intracellular Na^+^ or 10 mM Na^+^ and 30 *μ*M loxapine (*dark blue inverted triangle*). (C) Mean bar with SEM and individual data points for current density at +10 mV from the data presented in (*B*), with the same color representation.

Firstly, to replicate the importance of the previously proposed Na^+^ sensor, D839 was mutated to both glutamate and alanine. The D839E mutation, which lengthened the side chain without affecting the negative charge, gave currents that were qualitatively no different from WT K_Na_1.1. The current-voltage relationships closely resembled the WT K_Na_1.1 channel with 10 and 40 mM intracellular Na^+^, and no currents were recorded in 0 mM intracellular Na^+^ (Fig. 2 *B*). Consistent with Zhang and others (17), no currents were recorded in the presence of 0, 10, or 40 mM intracellular Na^+^ from D839A channels. Large, relatively voltage-independent currents were yielded upon perfusion of 30 *μ*M loxapine into the bath solution, confirming the presence of this Na^+^-insensitive mutant K_Na_1.1 channel at the cell membrane (Fig. 2).

### The conformational change in the βN-αQ loop and D884 controls the activation state of K_Na_1.1 channels

**Figure 3:**
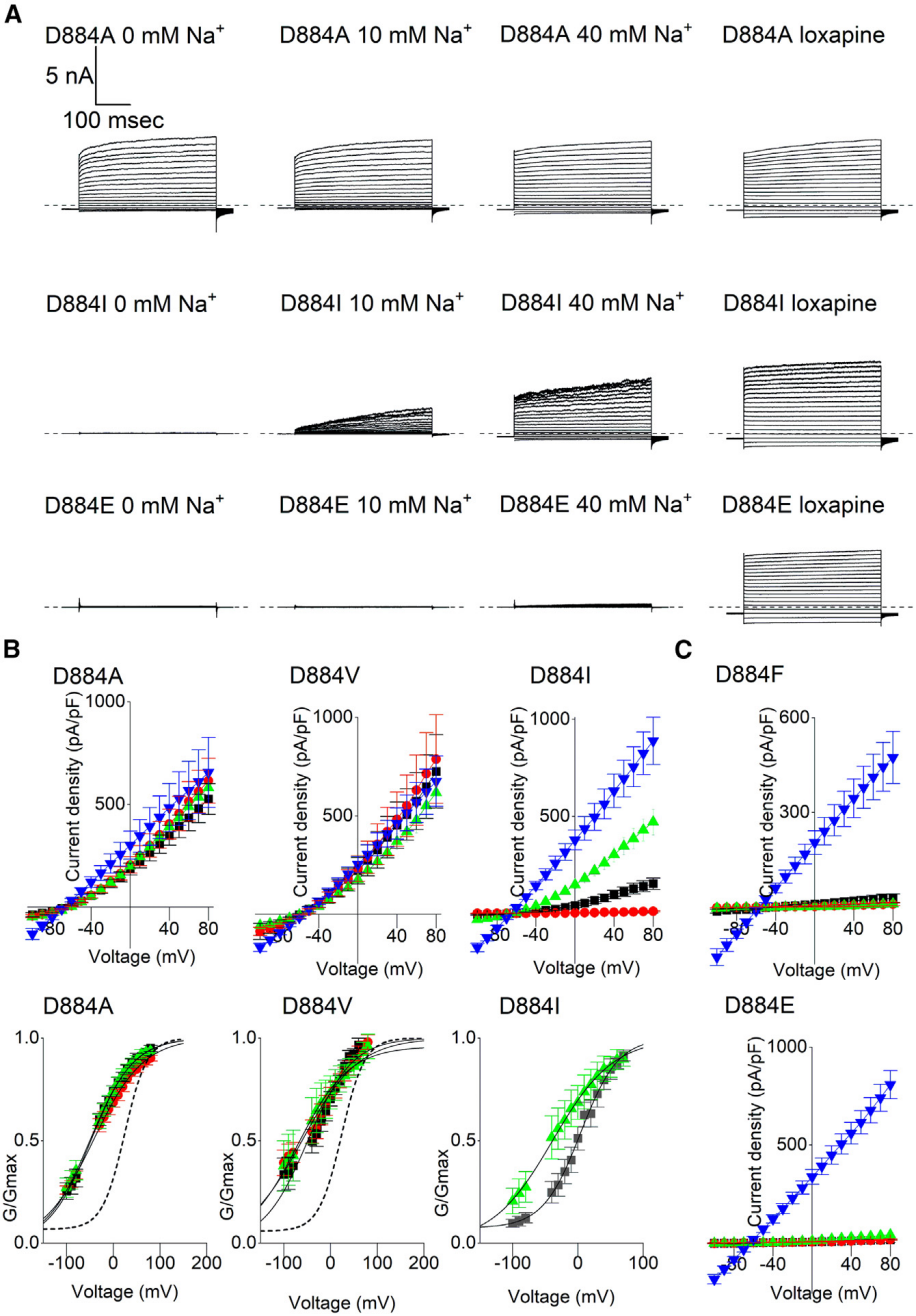
Role of D884 in controlling K_Na_1.1 channel activation. (*A*) Representative D884A, D884I, and D884E K_Na_1.1 whole-cell currents in response to 400 ms steps from −100 to +80 mV in 10 mV increments from a holding potential of −80 mV. The dashed lines indicate the zero-current level. (*B*) Mean ± SEM (*n* = 5–8 cells) current-voltage and conductance-voltage relationships for D884A, D884V, and D884I K_Na_1.1 with 0 (*red circle*), 10 (*black square*), and 40 (*green diamond*) mM intracellular Na^+^ or 10 mM Na^+^ and 30 *μ*M loxapine (*dark blue inverted triangle*). The mean conductance-voltage relationship for WT K_Na_1.1 recorded with 10 mM intracellular Na^+^ is indicated by a dotted line for comparison. (*C*) Mean ± SEM current-voltage relationships for D884F and D884E K_Na_1.1 with 0 (*red circle*), 10 (*black square*), and 40 (*green triangle*) mM intracellular Na^+^ or 10 mM Na^+^ and 30 *μ*M loxapine (*dark blue inverted triangle*).

The analysis of protein structures suggested that the movement of the D884 through the rotation of the βN-αQ loop could be a key step in the process of K_Na_1.1 activation by Na^+^. Alternatively, through its negative charge, it could be a candidate for Na^+^ binding, so we mutated D884 to alanine. Unexpectedly, this resulted in a large increase in channel activity and apparent loss of Na^+^ dependence. The voltage-dependent currents recorded from CHO cells expressing D884A K_Na_1.1 with 0, 10, or 40 mM intracellular Na^+^ were all similar (Figs. 3 *A* and S2 *A*). Perfusion of 30 *μ*M loxapine to cells had little effect on the current amplitude apart from an apparent loss of voltage dependence, as indicated by a straightening of the current-voltage relationship (Fig. 3 *B*). Mutation of the same residue to valine, which has a longer side chain by just one carbon, had similar effects (Fig. 3). Activation midpoints derived from conductance-voltage relationships fitted with a Boltzmann equation for D884A and D884V in 10 mM Na^+^ were significantly hyperpolarized compared to WT K_Na_1.1 (Table 1). With 40 mM intracellular Na^+^, the activation midpoints for D884A and D884V K_Na_1.1 were not significantly different from WT K_Na_1.1 under the same conditions. While WT K_Na_1.1 currents were negligible with 0 mM intracellular Na^+^, the activation midpoints for D884A and D884V with 0 mM Na^+^ were not significantly different from those obtained with 10 and 40 mM Na^+^ (Table 1).

**Table 1:** Parameters derived from Boltzmann fit of WT and D884 mutant K_Na_1.1 channel conductance with 0, 10, and 40 mM intracellular Na^+^

K_Na_1.1 variant	[Na^+^]_i_ (mM)	*V*_*0.5*_ (mV)	*z*
WT	10	2.15 ± 3.69	0.81 ± 0.09
WT	40	−46.63 ± 8.06	0.75 ± 0.14
D884A	0	−60.28 ± 9.60	0.47 ± 0.03
D884A	10	−61.92 ± 6.77^a^	0.60 ± 0.07
D884A	40	−60.33 ± 8.36	0.59 ± 0.09
D884V	0	−46.99 ± 11.08	0.61 ± 0.07
D884V	10	−61.08 ± 3.23^b^	0.57 ± 0.04
D884V	40	−60.86 ± 4.89	0.57 ± 0.08
D884I	10	0.23 ± 6.78	0.92 ± 0.02
D884I	40	−39.17 ± 14.32	0.66 ± 0.09

Data are presented as mean ± SEM (*n* = 5–9 cells). *V*_*0.5*_, half-maximal activation voltage.

a *p* < 0.0005 compared to WT *V*_*0.5*_ with 10 mM intracellular Na^+^ (independent one-way ANOVA with Tukey’s post hoc test). *z* was derived from the slope of the Boltzmann curve, *RT*/*zF*. No significant differences in *z* were found with mutant K_Na_1.1 compared to WT with either 10 or 40 mM intracellular Na^+^ (ANOVA).

b *p* < 0.005.

Upon increasing the size of the hydrophobic sidechain further by mutating D884 to isoleucine and phenylalanine, different effects were observed. D884I K_Na_1.1 currents were similar to those obtained from WT K_Na_1.1 with each intracellular Na^+^ concentration tested (Figs. 3 *B* and S2 *A*; Table 1). In contrast, no discernible current could be recorded from D884F K_Na_1.1 with any of the Na^+^ concentrations tested up to 40 mM, though function was rescued by loxapine application (Fig. 3 *C*). Finally, we mutated D884 to glutamate, which retained the negative charge but increased the side chain by one carbon. The D884E mutation in K_Na_1.1 had an effect similar to D884F, with negligible currents with 0, 10, and 40 mM intracellular Na^+^ each, but large whole-cell currents were recorded following the application of loxapine (Figs. 3, *A–C*, and S2 *A*).

**Figure 4:**
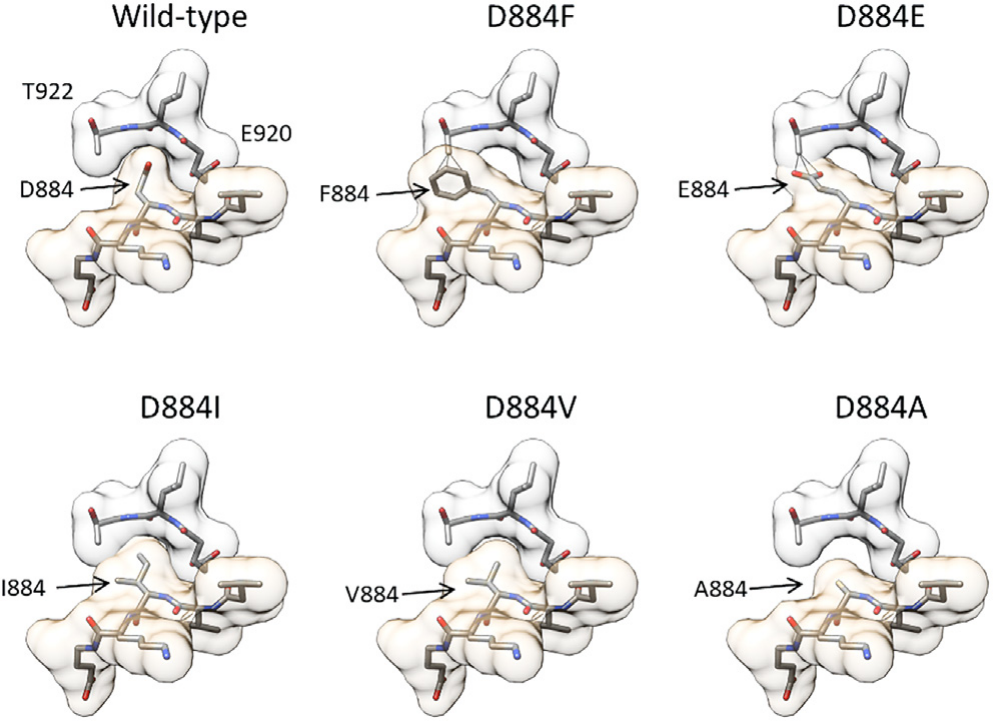
Molecular modeling of D884 mutations. Models were generated from the structure of chicken K_Na_1.1 in the Na^+^-activated state (PDB: 5U70), here with the numbering of conserved residues in human K_Na_1.1. In each model, the pocket formed by ^920^ELT^922^ in the βO strand is shown with pale gray space fill, with ^882^VVDKE^886^ in the βN-αQ loop with pale gold space fill below. With the D884F and D884E substitutions, predicted atomic clashes between the substituted side chains and T922 are indicated by solid black lines. No clashes were predicted in the other models.

These results suggested that it could be the size and not the charge of the side chain at amino acid position 884 that determined whether the K_Na_1.1 channel remained Na^+^ dependent or became locked in either the inactive or Na^+^-activated state under these conditions. We returned to the structure of the Na^+^-activated K_Na_1.1 channel and modeled the D884 mutations in silico. While aspartate and isoleucine at position 884 could be closely accommodated in the surrounding βO pocket, together with the smaller alanine and valine side chains with some leeway, substituting the larger glutamate and phenylalanine side chains resulted in steric clashes with T922 (Fig. 4). Additional energy minimization steps with the D884I model did not result in any discernable changes in orientation involving these side chains (Fig. S3 *A*). D884 was mutated to asparagine in rat K_Na_1.1 by Xu and others (21), resulting in Na^+^ insensitivity, and we found that this mutation could also result in a steric clash in silico (Fig. S3 *B*).

### The role of K885 and residues in or near the βO strand in Na^+^ activation

**Figure 5:**
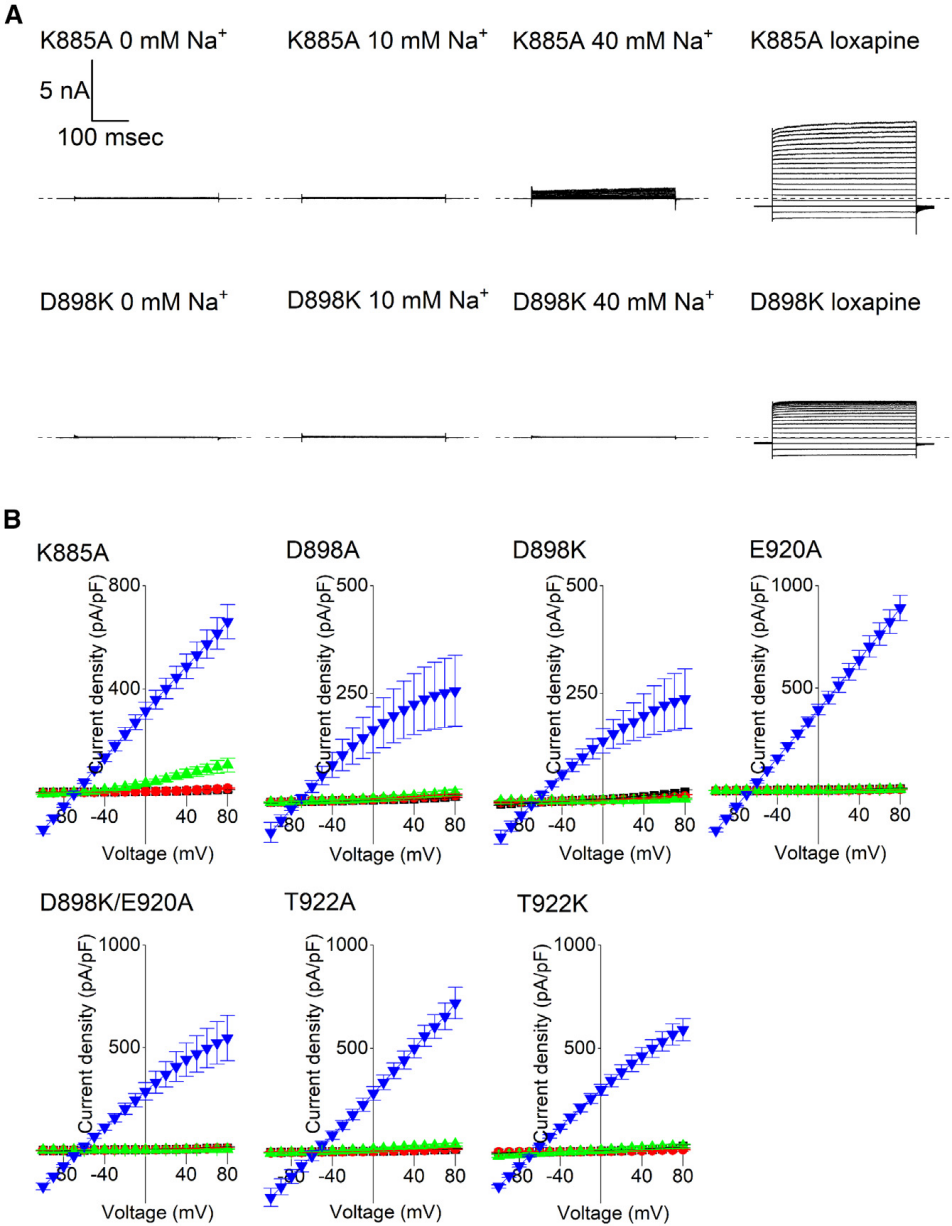
Disruption of Na^+^ activation through loss of a potential D839/K885 salt bridge and residues in or near βO. (*A*) Representative whole-cell K885A and D898K K_Na_1.1 currents in each condition, as indicated, in response to 400 ms steps from −100 to +80 mV in 10 mV increments from a holding potential of −80 mV. The dashed lines indicate the zero-current level. (*B*) Mean (±SEM, *n* = 5–8 cells) current-voltage relationships for mutated K_Na_1.1 channels in the presence of 0 (*red circle*), 10 (*black square*), and 40 (*green triangle*) mM intracellular Na^+^ or 10 mM Na^+^ and 30 *μ*M loxapine (*dark blue inverted triangle*).

The structural analysis conducted above suggested that Na^+^ activation causes the βN-αQ loop to transition from a disordered state to a conformation that could potentially be stabilized by a salt bridge between D839 (studied above) and K885. To test this idea, K885 was neutralized to alanine. Like with D839A K_Na_1.1, negligible currents were recorded from cells expressing K885A K_Na_1.1 with 0 and 10 mM intracellular Na^+^ (Figs. 5 and S2 *B*). A small current was recorded from K885A K_Na_1.1 when intracellular Na^+^ was elevated to 40 mM, suggesting a substantial decrease in Na^+^ sensitivity of the channel. Functional expression of K885A K_Na_1.1 channels was again confirmed by the addition of 30 *μ*M loxapine, which evoked large whole-cell currents comparable with those we observed earlier in the study.

We then questioned if the negatively charged (D898 and E920) or polar (T922) side chains in the vicinity of the pocket accommodating D884 in the activated state played a role in activation by Na^+^. Mutation of each of these to alanine similarly disrupted Na^+^ activation, with no significant currents recorded with 0, 10, or 40 mM intracellular Na^+^ (Figs. 5 and S2 *B*). Functional rescue was achieved with each of these mutant K_Na_1.1 channels following the application of 30 *μ*M loxapine. We initially considered whether these residues might contribute to a site for Na^+^ to occupy and activate the channel. To see if we could rescue one of these mutations, E920A, or attract D884 into this site and increase activity, D898 and T922 were mutated to the positively charged lysine. However, with each of the D898K, E920A/D898K, and T922K K_Na_1.1 channels, the effect was same as the alanine substitutions in this pocket (Figs. 5 and S2 *B*). Again, no currents were recorded with 0, 10, or 40 mM intracellular Na^+^, and functional rescue was achieved with 30 *μ*M loxapine application. We found that mutation of D898 to either alanine or lysine appeared to result in a weak inward rectification in loxapine-evoked current-voltage relationships (Fig. 5 *B*).

## Discussion

Our data suggest that the βN-αQ loop in RCK2 of K_Na_1.1 acts as a molecular switch controlling transitions between the Na^+^-activated and Na^+^-free inactive states. In the activated state, this loop may be stabilized by a salt bridge between K885 and D839. This explains why previous mutations of this aspartate, D812 in rat K_Na_1.1, by Zhang and others (17) resulted in a loss of Na^+^ sensitivity, leading to their proposal that this formed a Na^+^-binding site. Concurrently, the ability of D884 to occupy a pocket formed by the βO strand in the activated state appears to be important for activation. Mutating this side chain to either phenylalanine or glutamate prevented K_Na_1.1 channel activation by 40 mM intracellular Na^+^, but mutation to either alanine or valine caused the channel to adopt a constitutively activated, but voltage-gated, Na^+^-independent state. This could imply that the conformation adopted by this pocket in the absence of Na^+^ prevents D884 and the βN-αQ loop from adopting the stabilized state but can accommodate alanine and valine side chains in place of D884. This domain is highly conserved between K_Na_1.1 and K_Na_1.2, including each of the residues described here (Fig. 1 *A*), indicating a common mechanism.

One possible explanation for the different effects of the mutations relates solely to the size of the side chain at position D884, as considered in the in silico mutagenesis (Fig. 4). However, the amino acid substitutions may have wider-reaching effects on the protein structure through changes to the electrostatics in the locality of this region. Rather than promoting (D884A, D884V) or preventing (D884F, D884E) the adoption of the Na^+^-activated conformation, converse effects of these mutations on the apo state might underlie these effects. Furthermore, given the near 180° rotation of the βN-αQ loop between these two states, the mutations may affect the intervening conformational dynamics.

D884 was recently proposed by both Xu and others (21) and Zhang and others (22) to contribute to a potential cation binding site. Mutating the equivalent of D884 in rat K_Na_1.1 to asparagine rendered the channel nonfunctional at intracellular Na^+^ concentrations to up to 2 M (21). Consequently, and supported by molecular dynamics simulations, this aspartate was proposed as one of two Na^+^-binding sites in each subunit. In contrast, we found that the charge of this residue is not critical to K_Na_1.1 channel function, since D884I K_Na_1.1 channels behaved similarly to WT K_Na_1.1. In silico mutagenesis of this aspartate in the chicken K_Na_1.1 structure to asparagine caused a steric clash with acidic pocket residues, similar to D884E and D884F, substitutions of which prevented Na^+^ activation in our experiments. This explains the lack of activity with the asparagine mutation observed by Xu and others (21). D884 is part of cation-binding “site 2” identified by Zhang and others (22), who also studied the D884A K_Na_1.1 mutation. Using intracellular Na^+^ concentrations upwards from 100 mM, they found a fourfold decrease in the Na^+^ EC_50_. Since we recorded activated K_Na_1.1 channel currents in the absence of Na^+^, the residual Na^+^ dependence may arise from an alternative Na^+^-binding site with these higher concentrations. The anomalous density in cation site 2 of human K_Na_1.1 described by Zhang and others (22) was attributed to a K^+^ ion coordinated simultaneously by D839 and D884. This protein structure was obtained in Na^+^-free and KCl-rich conditions and attributed to the closed or inactive conformation. The structure of this region in the activated or open human K_Na_1.1 described in the same study is similar to that obtained with the chicken homolog (Fig. 1), where D884 is effectively replaced by K885 upon the conformational change in the βN-αQ loop, together with a loss of anomalous density in site 2. The significance of K^+^ binding to this site in the inactive conformation is not known, but it is important to note that it is vacated by D884 upon Na^+^ activation.

We are unable to propose a specific Na^+^-binding site from our investigation, but mutagenesis of residues D898, E920, and T922 that also disrupted Na^+^ activation would support the idea that Na^+^ binds in this region. These residues may have a role in coordinating Na^+^ ions, consistent with E920 having been identified by Xu and others (21) as a Na^+^-coordinating side chain, and may underlie the structural change required to accommodate D884 by the βO strand. Mutation of one of these residues, D898, to either lysine or alanine resulted in inward rectification of the K_Na_1.1 current-voltage relationship when activated by loxapine. It was noted in the structure of the chicken K_Na_1.1 channel that the ring of RCK domains forms a “funnel” that narrows as it approaches the pore-forming region and has a largely electronegative surface (19). D898 contributes to this highly negatively charged surface, which may contribute to ion conduction. The negatively charged residues are thought to attract K^+^ ions and contribute to the relatively high K^+^ conductance of this and other members of the channel subfamily, and mutation of D898 may have an effect of outward K^+^ currents, leading to the slight inward rectification observed.

Consistent with the model presented here, the rotation and adoption of a stable α-helix by the βN-αQ loop in RCK2 in the activated, but disordered in the Na^+^-free, K_Na_1.1 conformation were highlighted in the recently published structure of human K_Na_1.1 (17). The authors argue that this conformational change influences interactions between RCK2 and RCK1 domains, resulting in the overall expansion that opens the channel gate. In conclusion, this structural and our functional data point to this loop as the key regulator of K_Na_ channel activation by Na^+^. The constitutive activity with the D884A and D884V mutant K_Na_1.1 in the absence of Na^+^ provide further clues as to how this channel is regulated. Given that these mutant channels, with the smaller side chain, are able to activate without Na^+^, this indicates that the WT channel is primed for activation but requires a small structural change caused by Na^+^ binding to enable the βN-αQ loop to adopt the activated state. It is this inactive but primed state that may be disrupted by inherited mutations that result in K_Na_1.1 gain of function.

## Data and code availability

The unique reagents generated during this study are available upon reasonable request to the corresponding author. No additional data are associated with this article.

## Author contributions

B.A.C., J.D.L., A.C.K., and S.P.M. designed the study. B.A.C. and J.D.L. performed research. B.A.C., J.D.L., A.C.K., S.P.M., and N.P. analyzed and interpreted data. B.A.C. and J.D.L. drafted the manuscript. B.A.C., J.D.L., A.C.K., S.P.M., and N.P. edited and approved the manuscript.

## Supporting material

Document S1. Table S1 and Figures S1–S3

Document S2. Article plus supporting material
