# Supplementary material for: A molecular switch in RCK2 triggers sodium-dependent activation of KNa1.1 (KCNT1) potassium channels
Source: Biophys J. 2024 Apr 10;123(14):2145–53. doi: 10.1016/j.bpj.2024.04.007 (PMC11309980; doi:10.1016/j.bpj.2024.04.007)
Supplement: Document S1. Table S1 and Figures S1–S3 [file mmc1.pdf]

Biophysical Journal, Volume *123*

Supplemental information

A molecular switch in RCK2 triggers sodium-dependent activation of K_{Na}1.1 (KCNT1) potassium channels

Bethan A. Cole, Antreas C. Kalli, Nadia Pilati, Stephen P. Muench, and Jonathan D. Lippiat

A molecular switch in RCK2 triggers sodium-dependent activation of K_{Na}1.1 (KCNT1) potassium channels

Bethan A. Cole,[1,5] Antreas C. Kalli,[2,3] Nadia Pilati,[4] Stephen P. Muench,[1,3] and Jonathan D. Lippiat[1]

[1]School of Biomedical Sciences, University of Leeds, Leeds LS2 9JT, UK.

[2]Leeds Institute of Cardiovascular and Metabolic Medicine, University of Leeds, Leeds LS2 9JT, UK.

[3]Astbury Centre for Structural and Molecular Biology, University of Leeds, Leeds LS2 9JT, UK.

[4]Autifony Srl, Padova, 35127, Italy.

[5]Present address: Nuffield Department of Clinical Neurosciences, University of Oxford, Oxford OX3 9DU, UK.

SUPPORTING MATERIAL

Table S1 Equivalent amino acid positions in K_{Na}1.1 channel subunits from the human clone used for electrophysiological experiments in this study and in cryo-EM (1), cryo-EM structures of chicken K_{Na}1.1 (2), and rat K_{Na}1.1 used in previous studies of Na^+-activation of K_{Na}1.1 (3, 4).

Human	Chicken	Rat
E392	E371	E373
D839	D812	D818
D884	D857	D863
K885	K858	K864
D898	D871	D877
E920	E893	E899
T922	T895	T901

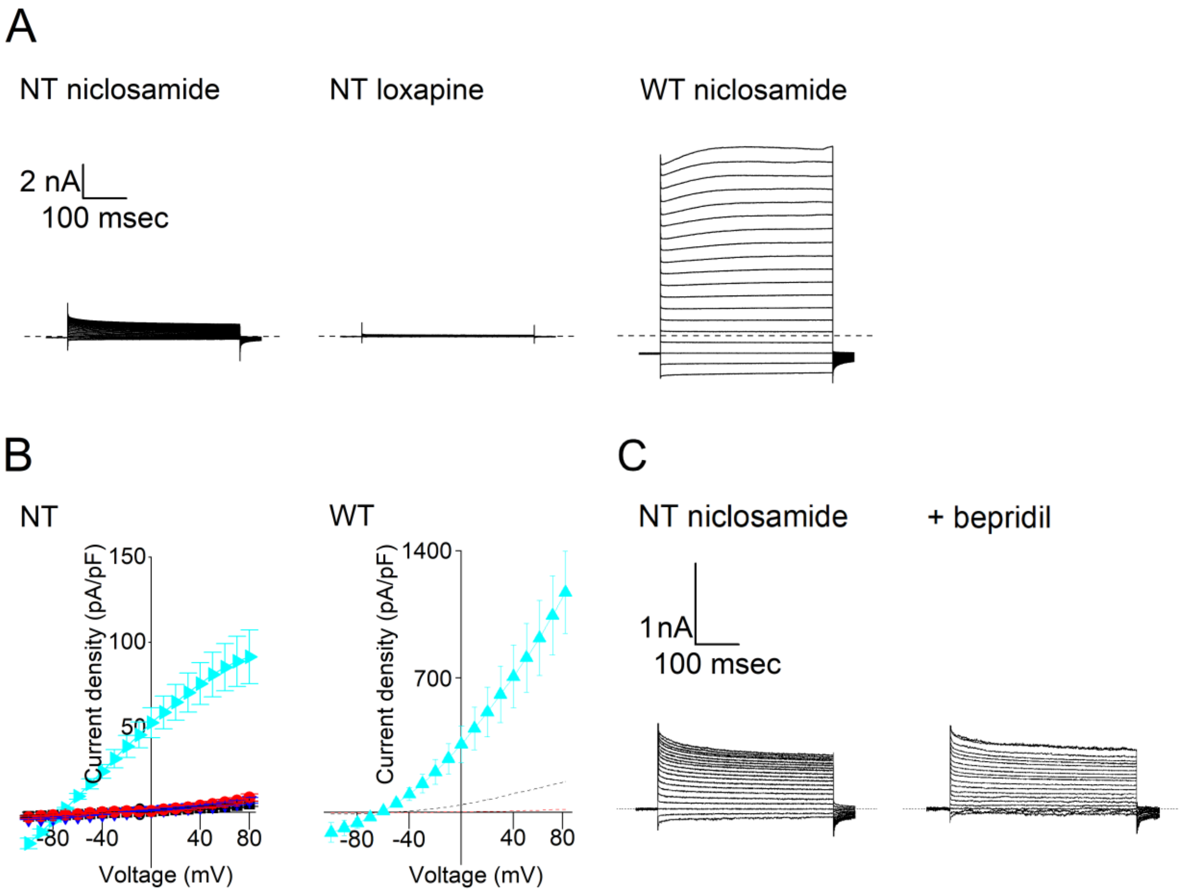

Figure S1. Evaluation of K_{Na}1.1 channel activators on control CHO cells. **A** Representative whole cell currents from non-transfected CHO cells (NT) and CHO cells transfected with WT human K_{Na}1.1 (WT), as indicated, in response to 400 ms steps from -100 to +80 mV in 10 mV increments from a holding potential of -80 mV. **B** Mean (± SEM, n=5 to 8 cells) current-voltage relationships for NT control in the presence of 0 (red ●), 10 (black ■) mM intracellular Na^+, 10 mM Na^+ + 30 µM niclosamide (cyan ▶) and 10 mM Na^+ + 30 µM loxapine (dark blue ▼), and WT K_{Na}1.1 channels in the presence of 30 µM niclosamide (cyan ▶) and 10 mM Na^+. WT K_{Na}1.1 current recorded with 10 mM intracellular Na^+ is indicated by a black dotted line, and 0 mM intracellular Na^+ is indicated by a red dotted line. **C** Representative niclosamide-activated whole cell currents from non-transfected CHO cells (NT) in response to 400 ms steps from -100 to +80 mV in 10 mV increments from a holding potential of -80 mV, before (left) and after (right) application of 10 µM bepridil.

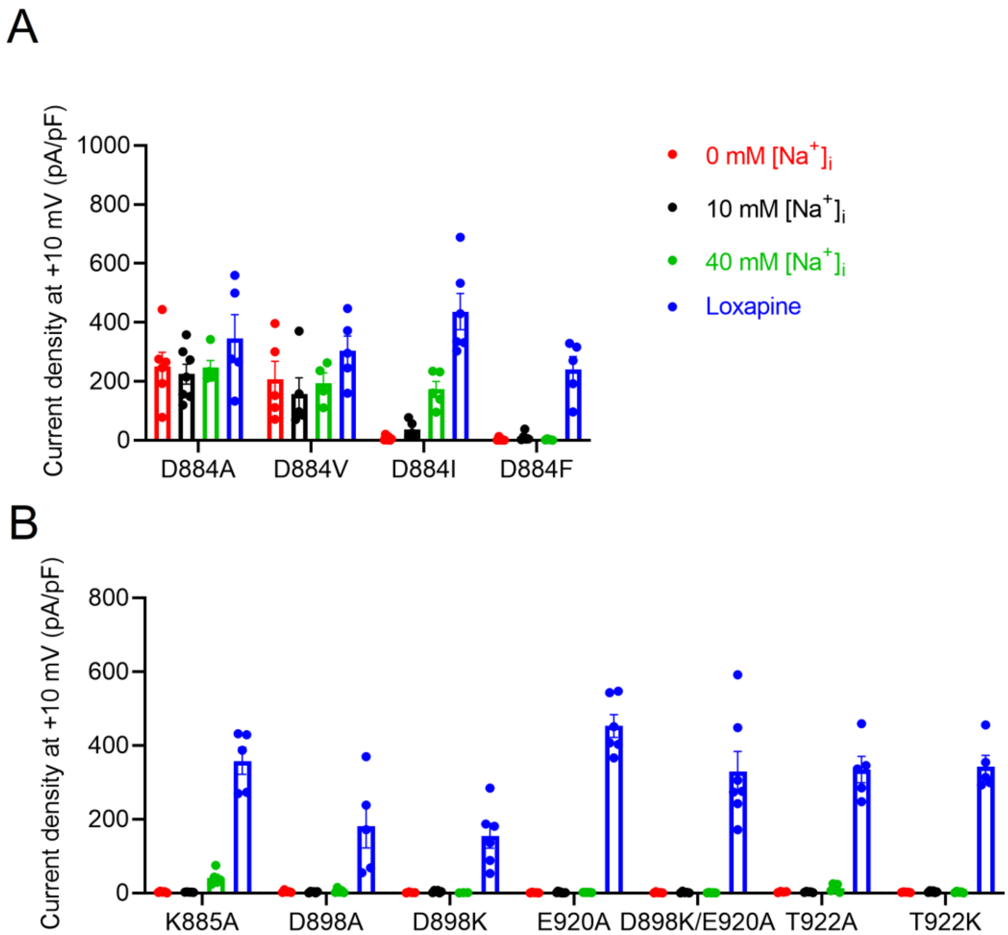

Figure S2. **Distribution of current densities obtained with mutant K_{Na}1.1.** Mean bar with SEM and individual data points representing current densities recorded at +10 mV for the mutant K_{Na}1.1 presented in main Figure 3 (**A**), and main Figure 5 (**B**).

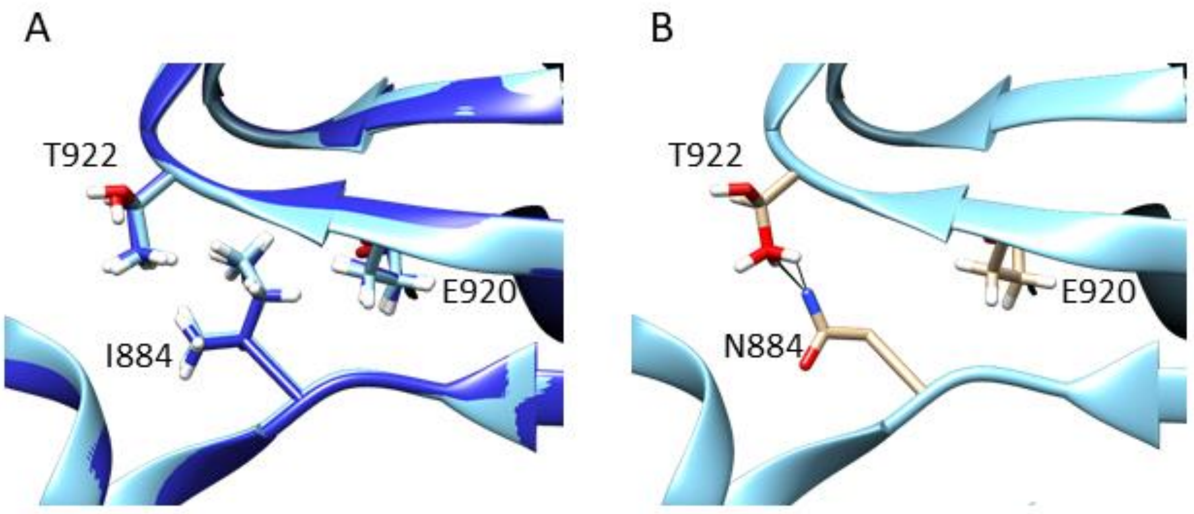

Figure S3. Further in silico modeling of K_{Na}1.1 D884. A Substitution of D884 at the equivalent position in activated chicken K_{Na}1.1 (PDB: 5U70) with isoleucine before (light blue) and after (dark blue) energy minimization in UCSF Chimera v1.13 (function “Minimize Structure” was used, using default step settings and no fixed atoms). **B** Substitution of D884 in the same model with asparagine (N884). Atomic clashes with T922 are shown by the black lines.

SUPPORTING REFERENCES

1. Zhang, J., S. Liu, J. Fan, R. Yan, B. Huang, F. Zhou, T. Yuan, J. Gong, Z. Huang, and D. Jiang. 2023. Structural basis of human Slo2.2 channel gating and modulation. Cell Reports 42, 112858.
2. Hite, R. K., and R. MacKinnon. 2017. Structural Titration of Slo2.2, a Na(+)-Dependent K(+) Channel. Cell 168, 390-399 e311.
3. Zhang, Z., A. Rosenhouse-Dantsker, Q. Y. Tang, S. Noskov, and D. E. Logothetis. 2010. The RCK2 domain uses a coordination site present in Kir channels to confer sodium sensitivity to Slo2.2 channels. J Neurosci 30, 7554-7562.
4. Xu, J., Y.-T. Lv, X.-Y. Zhao, J.-J. Wang, Z.-S. Shen, J. Li, F.-F. Zhang, J. Liu, X.-H. Wang, Y. Xu, Q. Geng, Y.-T. Ding, J.-J. Xu, M.-J. Tan, Z.-X. Li, R. Wang, J. Chen, W. Sun, M. Cui, D. E. Logothetis, J.-l. Cao, Q.-Y. Tang, and Z. Zhang. 2023. Identification of Sodium- and Chloride-Sensitive Sites in the Slack Channel. J Neurosi 43, 2665-2681.
